# Adult Attention-Deficit/Hyperactivity Disorder and the Risk of Dementia

**DOI:** 10.1001/jamanetworkopen.2023.38088

**Published:** 2023-10-17

**Authors:** Stephen Z. Levine, Anat Rotstein, Arad Kodesh, Sven Sandin, Brian K. Lee, Galit Weinstein, Michal Schnaider Beeri, Abraham Reichenberg

**Affiliations:** 1 School of Public Health, University of Haifa, Haifa, Israel; 2 Department of Gerontology, University of Haifa, Haifa, Israel; 3 Mental Health, Meuhedet Health Services, Tel Aviv, Israel; 4 Department of Community Mental Health, University of Haifa, Haifa, Israel; 5 Department of Psychiatry, Icahn School of Medicine at Mount Sinai, New York, New York; 6 Department of Medical Epidemiology and Biostatistics, Karolinska Institutet, Stockholm, Sweden; 7 Dornsife School of Public Health, Drexel University, Philadelphia, Pennsylvania; 8 The Herbert and Jacqueline Krieger Klein Alzheimer’s Research Center, Brain Health Institute, Rutgers University, Piscataway, New Jersey

## Abstract

**Importance:**

Evidence that adult attention-deficit/hyperactivity disorder (ADHD) is associated with an increased risk of dementia is scarce and inconsistent, and potential sources of bias are untested.

**Objective:**

To examine the association between adult ADHD and the risk of dementia.

**Design, Setting, and Participants:**

This prospective national cohort study consisted of 109 218 members of a nonprofit Israeli health maintenance organization born between 1933 and 1952 who entered the cohort on January 1, 2003, without an ADHD or dementia diagnosis and were followed up to February 28, 2020. Participants were aged 51 to 70 years in 2003. Statistical analysis was conducted from December 2022 to August 2023.

**Exposure:**

Adult ADHD was a time-varying covariate, classified as present from the age of the first diagnosis (using the *International Classification of Diseases, Ninth Revision*, and the *International Statistical Classification of Diseases and Related Health Problems, Tenth Revision*); otherwise, absent.

**Main Outcome and Measures:**

Cox regression models were fitted to quantify the association between adult ADHD and the risk of incident dementia with hazard ratios (HRs) and their 95% CIs unadjusted and in the primary analysis, using inverse probability weights, adjusted for 18 sources of potential confounding. In 14 complementary analyses, subgroup and sensitivity analyses were implemented.

**Results:**

At the beginning of the follow-up, the sample of 109 218 participants had a mean (SD) age of 57.7 (5.5) years, 56 474 participants (51.7%) were female, and 52 744 (48.3%) were male. During follow-up, 730 participants (0.7%) received a diagnosis of adult ADHD, and 7726 (7.1%) received a diagnosis of dementia. Dementia occurred among 96 of 730 participants (13.2%) with adult ADHD and 7630 of 108 488 participants (7.0%) without adult ADHD. In the primary analysis, compared with the absence of adult ADHD, the presence of adult ADHD was statistically significantly (*P* < .001) associated with an increased dementia risk (unadjusted HR, 3.62 [95% CI, 2.92-4.49; *P* < .001]; adjusted HR, 2.77 [95% CI, 2.11-3.63; *P* < .001]). Twelve of the 14 complementary analyses did not attenuate the conclusions based on the results of the primary analysis. There was, however, no clear increase in the risk of dementia associated with adult ADHD among those who received psychostimulant medication, and evidence of reverse causation was mild.

**Conclusions and Relevance:**

In this cohort study of individuals born between 1933 and 1952 and followed up in old age, adult ADHD was associated with an increased risk of dementia. Policy makers, caregivers, patients, and clinicians may wish to monitor reliably for ADHD in old age.

## Key Points

**Question:**

Is adult attention-deficit/hyperactivity disorder (ADHD) associated with an increased risk of dementia?

**Findings:**

In this national birth cohort study, 109 218 participants with or without a diagnosis of adult ADHD were followed up for 17.2 years for dementia. The presence of adult ADHD was statistically significantly associated with an increased risk of dementia.

**Meaning:**

This study suggests that adult ADHD is associated with an increased risk of dementia and warrants reliable assessment in adulthood.

## Teaser

This cohort study examines the association between adult attention-deficit/hyperactivity disorder and the risk of dementia.

## Introduction

Dementia is a syndrome characterized by dysfunction in daily life due to cognitive impairment.^1^ It ranks as a leading cause of disability and mortality.^2^ It is estimated that in 2022, among US individuals aged 65 years or older, 6.5 million had dementia, a figure that is forecasted to increase to 13.8 million by 2060.^2^ Hence, identifying risk and preventive factors for dementia is an international priority.^3,4^

Although generally defined as a neurodevelopmental disorder, evidence supports the concept of adult-onset attention-deficit/hyperactivity disorder (ADHD).^5^ Research has reported that 5% of children with ADHD meet ADHD criteria in adulthood, constituting 3% of adult ADHD cases.^5^ Studies also show that child and adult ADHD present different social, psychological, and genetic profiles.^5,6,7^ Despite being distinct from childhood ADHD, little is known about adult ADHD.^5^

Adult ADHD may be associated with an increased risk of dementia based on common health outcomes, clinical observations, family-based research, and epidemiologic risk studies. Meta-analyses have identified 6 health outcomes (ie, depression, midlife hypertension, smoking, type 2 diabetes, and low levels of education and physical activity)^8^ that are modifiable dementia risk factors^9^ and consequences of ADHD.^10,11^ Clinical observations and research suggest that adult ADHD appears to mimic some cognitive symptoms of dementia (eg, memory loss).^12^ Nonetheless, ADHD is underascertained in specialist old age clinics with a dementia focus.^13^ Family-based research demonstrates that ADHD is associated with dementia across generations, but the magnitude of the association is attenuated by less genetic kinship, suggesting shared familial risk between the diagnoses.^8^ Most,^14,15,16,17^ but not all,^18^ epidemiologic studies support a significant association between ADHD and the risk of dementia. However, the association is null in some studies^18^ and is stronger among males than females.^17^

The association between adult ADHD and dementia risk remains a topic of interest because of inconsistent results and key factors are yet to be studied. These factors include prescribed psychostimulant medications and reverse causation. Psychostimulant medications are cognitive enhancers used to treat ADHD and so may modify the potential trajectory of cognitive impairment.^19^ Reverse causation challenges the association between adult ADHD and dementia because adult ADHD is accompanied by cognitive impairments that resemble dementia and coincide with the onset of the protracted preclinical phase of dementia (eFigure 1 in Supplement 1). The present study aims to examine the association between adult ADHD and the risk of incident dementia.

## Methods

### Population

The present cohort study source population was ascertained from electronic health records held at Meuhedet Healthcare Services (hereafter referred to as *Meuhedet*), a nonprofit health maintenance organization (HMO) that provides health care services with national coverage to 14% of the total population of Israel.^20^ In brief, it is illegal for nonprofit HMOs in Israel to refuse membership based on demographic factors, health conditions, or medication needs, thereby limiting sample selection bias in the present study. These data at Meuhedet include every patient with a clinical diagnosis of dementia since 2000. The institutional review board at the University of Haifa and the Meuhedet-associated Helsinki institutional review board granted ethical approval to conduct the present study with a waiver of written informed consent because the data were deidentified. This study followed the Strengthening the Reporting of Observational Studies in Epidemiology (STROBE) reporting guideline.

### Study Design

**Figure 1.**
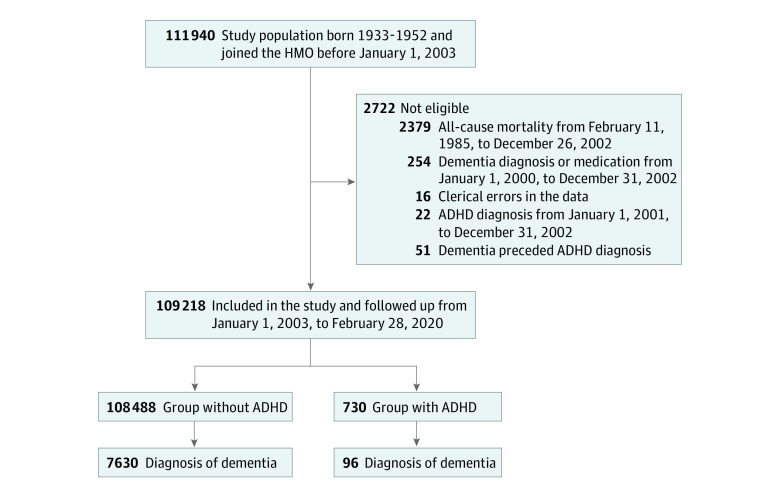
Study Flow Diagram Study flow diagram of cohort selection, inclusion, exclusion, and follow-up. ADHD indicates attention-deficit/hyperactivity disorder; HMO, health maintenance organization.

A prospective birth cohort study design was implemented. The eligible sample consisted of Israeli citizens who were nationwide Meuhedet members born between 1933 and 1952. Participants with a diagnosis of or medication for dementia or diagnosis of ADHD by December 31, 2002, were not eligible for inclusion (Figure 1). The cohort was followed up from January 1, 2003 (mean [SD] age, 57.7 [5.5] years), to death, leaving the HMO, dementia, or the end of the study on February 28, 2020 (mean [SD] age, 75.5 [6.0] years), whichever came first, for the risk of dementia. February 28, 2020, was chosen as the end of follow-up because the COVID-19 pandemic that began in May 2020 in Israel may have affected the study variable rates.

### Outcome: Ascertainment of Incident Dementia

The Meuhdet diagnoses of dementia are based on the *International Classification of Diseases, Ninth Revision* (*ICD-9*)^21^ (codes 331.0-331.9) and the *International Statistical Classification of Diseases and Related Health Problems, Tenth Revision* (*ICD-10*)^22^ (codes F00-F03). The estimated prevalence of dementia in this HMO is 6.6%,^23^ which resembles estimates in Western Europe (6.9%) and the US (6.5%).^24^ The diagnosis of dementia is ascertained by geriatricians, neurologists, or psychiatrists and has been used in previous descriptive^23^ and analytic^25^ epidemiologic research.

### Exposure: Ascertainment of Adult ADHD

Diagnoses of ADHD were ascertained based on *ICD-9* (code 314) or *ICD-10* (code F90) codes from January 1, 2003, to February 28, 2020. Each ADHD diagnosis is assigned by a psychiatrist, neurologist, or clinical neuropsychologist, all of whom are board certified. An ADHD diagnosis may also be assigned by a board-certified pediatrician, general practitioner, or family physician, who must undergo a Ministry of Health–certified course on ADHD diagnosis. An ADHD diagnosis requires a standardized neuropsychological assessment, typically done in testing centers specializing in ADHD.

### Covariates

The study covariates were chosen to adjust for the possibility of confounding.^26^ These covariates were background information, comorbid health conditions, and use of stimulant medication. The background information was the age at the beginning of the study in 2003 (entered as linear and quadratic terms),^27^ sex (male or female), and neighborhood socioeconomic status (classified as low, medium, or high). Smoking status was a time-varying covariate that was classified as present from the report of smoking forward and otherwise classified as absent. Comorbid health conditions were all ascertained as time-dependent covariates and classified as present from the first diagnosis onward, otherwise classified as absent. These comorbid health conditions were depression, obesity, chronic obstructive pulmonary disease, hypertension, atrial fibrillation, heart failure, ischemic heart disease, cerebrovascular disease, diabetes, Parkinson disease, traumatic brain injury, migraine, and mild cognitive impairment (*ICD-9* and *ICD-10* codes are in eTable 1 in Supplement 1).

Psychostimulant medication was derived based on Anatomical Therapeutic Chemical (ACT) Classification System codes classified as psychostimulants, agents used for ADHD, and nootropics (ie, ACT code N06B).^28^ Prescribed psychostimulant medication purchases are available to individuals only through their HMO, dispensed by pharmacies nationwide, all heavily subsidized, and are continuously recorded in the HMO electronic health records. These psychostimulants were prescribed and purchased and were analyzed as a time-varying covariate for purchase intervals based on a minimum time window of 60 days and with a duration of at least 90 days.

### Statistical Analysis

Statistical analysis was conducted from December 2022 to August 2023. Participant characteristics were computed to characterize the source population and present differences between the group with and the group without adult ADHD for the study covariates. Next, rates per 10 000 person-years of follow-up were estimated.^29^

Cox proportional hazards regression models were fitted to estimate hazard ratios (HRs) for dementia risk, with ADHD as a time-varying covariate. All HRs were presented with the associated 2-sided 95% CIs, using robust SEs.^30^ All models were implemented with age as the underlying timescale.^31^ Inverse probability weights were used in the adjusted models to address residual confounding.^32^ This approach incorporated time-independent and time-dependent covariates. To generate the inverse probability weights, the covariates of the primary model were included with ADHD as the outcome. The adjusted models included all the study covariates and inverse probability weights to improve the control of confounding. Also, the age-sex distribution was calculated in the study sample as the proportion of the Israeli national population, and the analysis was weighted to represent the entire population of Israel.^33^ Each participant was followed up for a dementia diagnosis, leaving the HMO, all-cause mortality, or the end of follow-up, whichever came first (eFigure 2 in Supplement 1).

The groups with or without adult ADHD were contrasted unadjusted (with only the binary covariate of ADHD), and then in the primary model adjusted for the study covariates. For the unadjusted model, we estimated the cumulative incidence proportion^34^ and plotted the cumulative incidence curves to present the risk of incident dementia for the groups with or without adult ADHD. The proportional hazards assumption was tested with the standard statistical test for the Cox proportional hazards regression model.^34^ The covariates of age at cohort entry (linear and quadratic), sex, and socioeconomic status were not time-varying covariates, whereas smoking, comorbid health conditions, ADHD medication, and the diagnosis of ADHD were time-dependent covariates. Models were fitted in R, version 4.1 (R Project for Statistical Computing), using the survival^34^ and ipw libraries.^32^ All *P* values were from 2-sided tests, and results were deemed statistically significant at *P* < .05.

The robustness of the results from the primary analysis was challenged in 14 complementary analyses. To consider subgroups, owing to possibly differential dementia risks, the primary analytic model was refitted to examine the ADHD interactions with sex (model 1)^17^ and smoking status (model 2) and was restricted to different age groups at the beginning of the study: aged 60 years or younger (model 3) and older than 60 years (model 4). In a sensitivity analysis, we examined the risk of dementia up to and then older than 65 years of age separately to distinguish between early-onset dementia (model 5) and late-onset dementia (model 6).^3^

Next, we examined aspects of ADHD in sensitivity analyses. To consider diagnostic reliability, we compared the group without ADHD with individuals with 1 adult ADHD diagnosis (model 7) and more than 1 adult ADHD diagnosis (model 8). We examined ADHD diagnosis as a static covariate (model 9), as ADHD is generally considered a neurodevelopmental disorder originating in early life. The adult ADHD interaction with psychostimulant medication exposure was examined (model 10). Among individuals with adult ADHD, groups exposed vs unexposed to psychostimulants were compared (model 11).

Finally, in sensitivity analysis, to examine reverse causation,^35^ the analysis was stratified by sequential durations of follow-up. Based on 3 successive 5-year intervals, the associations between ADHD and dementia risk were scrutinized (models 12-14). Attention-deficit/hyperactivity disorder was assumed to have a weaker association with dementia risk in the preclinical dementia stage when the assessment was performed long before the onset of dementia and to have a stronger association with dementia risk when the assessment was performed nearer to the dementia diagnosis. A stronger association in the later intervals rather than the earlier intervals of follow-up suggests that reverse causation occurs.

## Results

### Sample Characteristics

At the beginning of the follow-up, the sample of 109 218 participants had a mean (SD) age of 57.7 (5.5) years, 56 474 participants (51.7%) were female, and 52 744 participants (48.3%) were male (Table). The group with adult ADHD was younger than the group without adult ADHD (mean [SD] age, 55.7 [4.5] years vs 57.7 [5.5] years). Source population sample characteristics by the ADHD diagnostic status were computed (Table).

**Table.** Sample Characteristics

Variable classification	Total, No. (%) (N = 109 218)	ADHD unexposed, No. (%) (n = 108 488)	ADHD exposed, No. (%) (n = 730)
Dementia	7726 (7.1)	7630 (7.0)	96 (13.2)
Age in 2003, median (IQR), y	56.3 (53.0-62.0)	56.3 (53.0-62.0)	54.7 (52.0-58.5)
Sex			
Male	52 744 (48.3)	52 308 (48.2)	436 (59.7)
Female	56 474 (51.7)	56 180 (51.8)	294 (40.3)
SES			
Low	33 180 (30.4)	32 983 (30.4)	197 (27.0)
Medium	65 074 (59.6)	64 592 (59.5)	482 (66.0)
High	10 964 (10.0)	10 913 (10.1)	51 (7.0)
Smoker^a^			
Unexposed	86 905 (79.6)	86 430 (79.7)	475 (65.1)
Exposed	22 313 (20.4)	22 058 (20.3)	255 (34.9)
Depression^a^			
Unexposed	104 079 (95.3)	103 468 (95.4)	611 (83.7)
Exposed	5139 (4.7)	5020 (4.6)	119 (16.3)
Obesity^a^			
Unexposed	85 460 (78.2)	85 005 (78.4)	455 (62.3)
Exposed^a^	23 758 (21.8)	23 483 (21.6)	275 (37.7)
COPD^a^			
Unexposed	97 756 (89.5)	97 117 (89.5)	639 (87.5)
Exposed	11 462 (10.5)	11 371 (10.5)	91 (12.5)
Hypertension^a^			
Unexposed	44 787 (41.0)	44 537 (41.1)	250 (34.2)
Exposed	64 431 (59.0)	63 951 (58.9)	480 (65.8)
Atrial fibrillation^a^			
Unexposed	99 955 (91.5)	99 281 (91.5)	674 (92.3)
Exposed	9263 (8.5)	9207 (8.5)	56 (7.7)
Heart failure^a^			
Unexposed	105 416 (96.5)	104 704 (96.5)	712 (97.5)
Exposed	3802 (3.5)	3784 (3.5)	18 (2.5)
IHD^a^			
Unexposed	89 966 (82.4)	89 373 (82.4)	593 (81.2)
Exposed	19 252 (17.6)	19 115 (17.6)	137 (18.8)
Cerebrovascular disease^a^			
Unexposed	93 480 (85.6)	92 876 (85.6)	604 (82.7)
Exposed	15 738 (14.4)	15 612 (14.4)	126 (17.3)
Diabetes^a^			
Unexposed	79 993 (73.2)	79 484 (73.3)	509 (69.7)
Exposed	29 225 (26.8)	29 004 (26.7)	221 (30.3)
Parkinson disease^a^			
Unexposed	107 014 (98.0)	106 304 (98.0)	710 (97.3)
Exposed	2204 (2.0)	2184 (2.0)	20 (2.7)
TBI^a^			
Unexposed	108 377 (99.2)	107 657 (99.2)	720 (98.6)
Exposed	841 (0.8)	831 (0.8)	10 (1.4)
Migraine^a^			
Unexposed	103 557 (94.8)	102 906 (94.9)	651 (89.2)
Exposed	5661 (5.2)	5582 (5.1)	79 (10.8)
MCI^a^			
Unexposed	108 314 (99.2)	107 608 (99.2)	706 (96.7)
Exposed	904 (0.8)	880 (0.8)	24 (3.3)
Psychostimulant^a^			
Unexposed	107 868 (98.8)	107 301 (98.9)	567 (77.7)
Exposed	1350 (1.2)	1187 (1.1)	163 (22.3)

Abbreviations: ADHD, attention-deficit/hyperactivity disorder; COPD, chronic obstructive pulmonary disease; IHD, ischemic heart disease; MCI, mild cognitive impairment; SES, socioeconomic status; TBI, traumatic brain injury.

^a^ Exposure is at any time point during follow-up.

The follow-up period for incident dementia was 17.2 years. During follow-up, 730 participants (0.7%) received a diagnosis of adult ADHD, and 7726 (7.1%) received a diagnosis of dementia. A total of 163 of the 730 participants with ADHD (22.3%) received psychostimulant treatment (Table). The percentage of participants with an incident dementia diagnosis was 13.2% (96 of 730) among those classified with adult ADHD and 7.0% (7630 of 108 488) among those without adult ADHD (Figure 1). Rates of dementia per 10 000 person-years were estimated at 5.19 (95% CI, 4.20-6.34) for the group with adult ADHD and 1.44 (95% CI, 1.40-1.47) for the group without ADHD (eTable 2 in Supplement 1). The cumulative incidence curves showed that the group with an ADHD diagnosis had higher incident dementia rates compared with the group without an ADHD diagnosis. Among individuals with a diagnosis of ADHD, 42.9% (6 of 14) received a diagnosis of dementia at 85 years of age compared with 15.2% of individuals without ADHD (1223 of 8032). At 60 years of age, the corresponding figures were 1.6% (3 of 182) and 0.5% (382 of 72 116) (eFigure 3 in Supplement 1).

### ADHD and the Risk of Dementia

**Figure 2.**
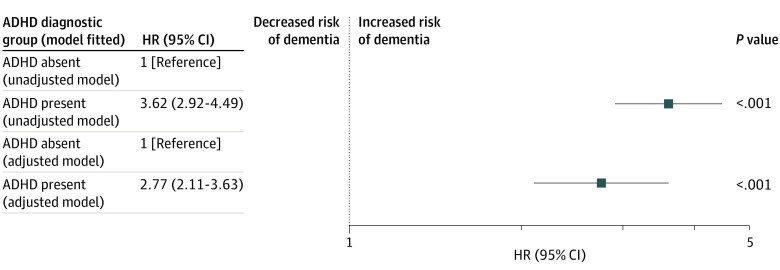
Primary Analysis of the Association Between Attention-Deficit/Hyperactivity Disorder (ADHD) and the Risk of Dementia HR indicates hazard ratio from the Cox proportional hazards regression model. 95% CIs are Wald 2-sided 95% CIs. *P* values are for test of the hypothesis HR = 1 vs the hypothesis HR ≠ 1.

The test of the departure from the proportional hazards assumption for the association between ADHD and the risk of incident dementia was not statistically significant (χ^2^ = 2.42; *P* = .12). In the primary analysis, the group with adult ADHD had a statistically significantly (*P* < .001) higher risk of incident dementia (unadjusted HR, 3.62 [95% CI, 2.92-4.49; *P* < .001]; adjusted HR, 2.77 [95% CI, 2.11-3.63; *P* < .001]; Figure 2) (adjusted model of all covariates is in eTable 3 in Supplement 1) compared with the group without ADHD.

### Complementary Analyses

**Figure 3.**
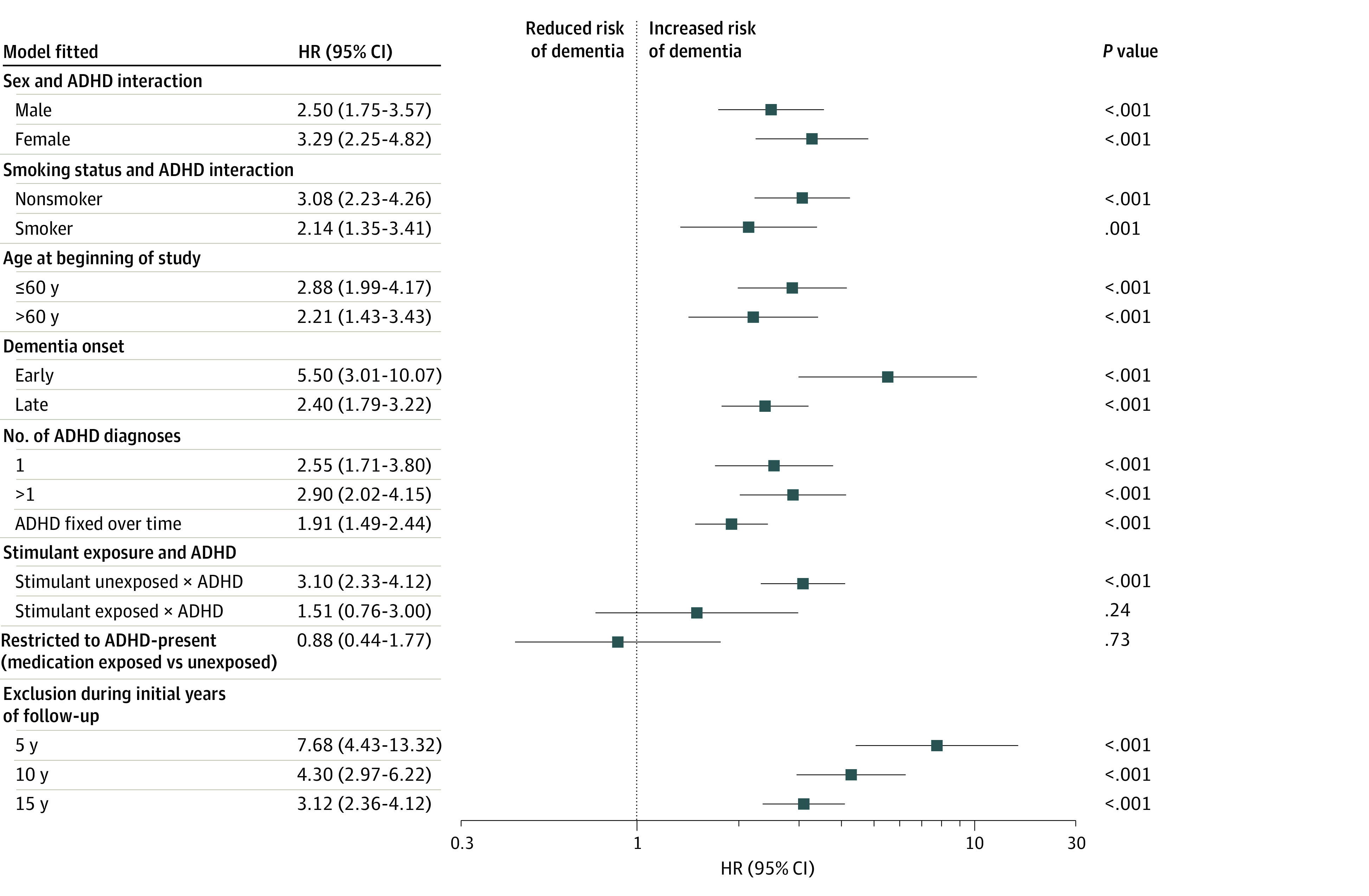
Complementary Analysis of the Association Between Attention-Deficit/Hyperactivity Disorder (ADHD) and the Risk of Dementia HR indicates hazard ratio from the Cox proportional hazards regression model. 95% CIs are Wald 2-sided 95% CIs. *P* values are for test of the hypothesis HR = 1 vs the hypothesis HR ≠ 1.

The robustness of the primary analysis was challenged in 14 complementary analyses by refitting the adjusted Cox proportional hazards regression model. The primary analytic model was refitted in subgroup analyses to examine the adult ADHD interactions with sex (model 1) and smoking status (model 2) and restricted by age at the beginning of the study to 60 years of age or younger (model 3) and older than 60 years of age (model 4). These results did not attenuate the conclusions of the primary results (Figure 3). In sensitivity analyses, results of modeling early-onset and late-onset dementia (models 5 and 6, respectively) did not attenuate the conclusions of the primary results. Sensitivity analysis of individuals without ADHD compared with those with a single and then more than 1 adult ADHD diagnosis (Figure 3; models 7 and 8, respectively) did not attenuate the conclusions based on the primary results. Diagnosis of ADHD as a static covariate was significantly associated with dementia risk, but the magnitude of the effect size was reduced compared with the primary analysis (Figure 3; model 9). There was no clear association between ADHD and dementia risk among those with psychostimulant medication exposure (Figure 3; models 10 and 11).

To examine reverse causation, the analysis was stratified by 3 sequential 5-year successive follow-up durations. The models did not attenuate the conclusion based on the results of the primary model. However, the closer the duration was to the dementia diagnosis, the higher the HR that quantified the association between ADHD and the risk of dementia (Figure 3; models 12-14).

## Discussion

This prospective cohort study examined the association between adult ADHD and dementia risk. The present study results showed that an adult ADHD diagnosis was associated with a 2.77-fold increased risk of incident dementia. Complementary analyses generally supported this association.

The present study finding that adult ADHD is associated with a higher dementia risk is consistent with most,^14,15,16,17^ but not all,^18^ prior epidemiologic studies. It may be plausible that adult ADHD reflects a brain pathobiological process that reduces the ability to compensate for the effects of later-life neurodegenerative and cerebrovascular processes. Less cognitive and brain reserve may result in pathobiological processes of ADHD that, in turn, reduce compensatory abilities. This explanation is consistent with our findings that show, for the first time to our knowledge, that the association between adult ADHD and dementia risk showed mild evidence of reverse causation.

There are different interpretations of the finding that adult ADHD treated with psychostimulant medication was not clearly associated with an increased dementia risk. It is possible that ADHD treated with medication reflects more severe ADHD compared with ADHD not treated with prescribed psychostimulants, which may reflect milder symptoms or even a less-accurate diagnosis. Also, ADHD is a chronic condition and may have negative long-term sequelae when untreated. Hence, the lack of association among individuals exposed to psychostimulants contradicts the conclusion that ADHD is associated with dementia risk. Alternatively, one may accept our ADHD ascertainment procedures. Among patients with adult ADHD, psychostimulant exposure is uncommon (22.3% in our study and less elsewhere^5^). The ADHD medication and diagnosis combination is 0.5% (567 of 109 218), possibly an underestimate of adult ADHD. Also, psychostimulants are cognitive enhancers hypothesized to reduce dementia risk.^19^ In addition, our study results should be interpreted within the broader literature that generally supports the association between adult ADHD and dementia.^14,15,16,17^ Seen this way, including psychostimulants in the association between adult ADHD and dementia risk may account for confounding by medication. That said, future research is necessary, including the assessment of adult ADHD symptom severity, to examine the role of psychostimulant medication in old age.

### Limitations

Our study has noteworthy limitations. We lacked information on childhood-onset ADHD. However, adult and childhood ADHD groups have different profiles,^5,6,7^ and epidemiologic evidence indicates that few instances of childhood ADHD are associated with adult ADHD.^5^ Hence, our findings aim to be relevant to adult ADHD and not childhood ADHD. Similarly, the rate of adult ADHD may be underascertained in the present study. The prevalence of adult ADHD in meta-analysis was estimated at 0.2% based on clinical diagnoses, 0.1% based on treatment rates, and 2.2% based on research diagnoses.^36^ Also, a recent epidemiological study of ADHD and dementia based on the strengths of the Swedish registry-based data classified 0.3% of the population with adult ADHD.^17^ Hence, the rate of adult ADHD of 0.7% based on clinical diagnoses that likely require clinical attention observed in the current data exceeds that found in other high-quality, registry-based studies of the prevalence^36^ and incidence^17^ of adult ADHD. Also, we were unable to examine the symptoms of ADHD (eg, inattention). In our study, we documented aspects that were potentially associated with the reliability of the ascertainment of ADHD. Namely, we pointed out the requirements of professional training, neuropsychological testing, and clinical impressions. In sensitivity analysis, we observed that the results were not attenuated by considering individuals with single or multiple ADHD diagnoses. Nonetheless, this study relies on clinical, not research, diagnoses.

There was no clear association between adult ADHD and dementia among individuals with ADHD who received psychostimulant medications. Due to the underdiagnosis of dementia as well as bidirectional misdiagnosis, this association requires further study before causal inference is plausible. There are multiple risks (eg, increased blood pressure or heart rate)^37^ and benefits (eg, reduced risk of accidents and injuries)^38^ to weigh before considering psychostimulant medication as a dementia prevention strategy. Future research is warranted to study the possible efficacy and adverse effects of psychostimulant medication in old age, before translation to the clinic can be considered.^12^

Dementia was most probably underascertained in our study. It seems, however, unlikely that the underascertainment of dementia would reduce the effect sizes of the study risk estimates to null. Also, the rate of dementia in our data resembles that in other nations.^24^ Attention-deficit/hyperactivity disorder may be associated with dementia owing to other factors (eg, apathy, apolipoprotein E epsilon 4 allele [APOE ε4]). However, the study covariates were chosen based on conditions that were previously found to be associated with ADHD and dementia (ie, met the definition of a confounder [unlike APOE ε4, which has not been associated with ADHD risk]).

In our data, it was not possible to assess lifelong low academic achievement and cognitive reserve. Also, we could not distinguish cognitive impairment due to lifelong low academic achievement that may result in an impaired cognitive reserve or due to incipient later-life neurodegenerative or cerebrovascular disease. Based on observational data, such as those in our study, causal inference is traditionally considered not possible. Nonetheless, we implemented inverse probability weighting to account for confounding bias. In addition, the increase in the association between ADHD and dementia at shorter lag times in the analysis of reverse causation suggests that there may be some diagnostic overlap. However, the reverse causation analyses did not attenuate the results of the primary analysis at any time interval.

## Conclusions

In this cohort study of 109 218 participants followed up to 17.2 years, after adjustment for 18 potential sources of confounding, the primary analysis indicated that an adult ADHD diagnosis was associated with a 2.77-fold increased dementia risk. Complementary analyses generally did not attenuate the conclusion of the primary analysis. This finding suggests that policy makers, caregivers, patients, and clinicians may wish to monitor ADHD in old age reliably.

## Notes

Supplement 1. **eTable 1.** Diagnoses in the Data **eTable 2.** Rates by 10,000 Person-Years **eTable 3.** Cox Proportional Hazards Regression Model From the Primary Analysis With All Study Covariates **eFigure 1.** Reverse Causation Illustrated **eFigure 2.** Follow-Up Time Illustration in the Survival Analysis **eFigure 3.** Cumulative Incidence Plot
eReferences.

Supplement 2.
Data Sharing Statement
