# Supplementary material for: Adult Attention-Deficit/Hyperactivity Disorder and the Risk of Dementia
Source: JAMA Netw Open. 2023 Oct 17;6(10):e2338088. doi: 10.1001/jamanetworkopen.2023.38088 (PMC10582792; doi:10.1001/jamanetworkopen.2023.38088)
Supplement: Supplement 1. — eTable 1. Diagnoses in the Data eTable 2. Rates by 10,000 Person-Years eTable 3. Cox Proportional Hazards Regression Model From the Primary Analysis With All Study Covariates eFigure 1. Reverse Causation Illustrated eFigure 2. Follow-Up Time Illustration in the Survival Analysis eFigure 3. Cumulative Incidence Plot eReferences. [file jamanetwopen-e2338088-s001.pdf]

Supplementary Online Content

Levine SZ, Rotstein A, Kodesh A, et al. Adult attention-deficit/hyperactivity disorder and the risk of dementia. *JAMA Netw Open.* 2023;6(10):e2338088. doi:10.1001/jamanetworkopen.2023.38088

eTable 1. Diagnoses in the Data
eTable 2. Rates per 10,000 Person-Years
eTable 3. Cox Proportional Hazards Regression Model From the Primary Analysis With All Study Covariates
eFigure 1. Reverse Causation Illustrated
eFigure 2. Follow-Up Time Illustration in the Survival Analysis
eFigure 3. Cumulative Incidence Plot
eReferences.

This supplementary material has been provided by the authors to give readers additional information about their work.

© 2023 Levine SZ et al. *JAMA Network Open.*

eTable 1. Diagnoses in the Data

Diagnosis	ICD codes used at index diagnosis (ICD-9 unless otherwise stated)
AF	427.31
Cerebrovascular	430, 430.9, 431, 431.9, 432, 432.1, 432.9, 433, 433.1, 433.2, 433.3, 433.9, 434, 434.1, 434.9, 434.91, 435, 435.1, 435.2, 435.9, 436, 436.9, 437, 437.1, 437.2, 437.3, 437.4, 437.5, 437.7, 437.9, 438
COPD	491, 491.1, 491.2, 491.8, 491.9, 492, 496
Depression	296.2, 296.21, 296.22, 296.23, 296.24, 296.25, 296.26, 296.3, 296.31, 296.32, 296.33, 296.34, 296.35, 296.36,<br>ICD-10: F32, F320, F3200, F3201, F321, F3210, F3211, F322, F323, F328, F329, F33, F3300, F3301, F331, F3310, F3311, F332, F333, F334, F338, F339, F34, F340, F341, F348, F349, F39
Diabetes	250, 250.01, 250.02, 250.03, 250.1, 250.11, 250.2, 250.3, 250.4, 250.41, 250.5, 250.51, 250.6, 250.61, 250.7, 250.71, 250.8, 250.9
HF	428, 428.1, 428.9
Hypertension	401, 401.1, 401.2, 401.9, 402, 402.01, 402.1, 402.11, 402.9, 402.91, 403, 403.1, 403.9, 404, 404.1, 404.9, 405, 405.01, 405.09, 405.1, 405.11, 405.9, 405.91, 405.99
IHD	410, 410.1, 410.2, 410.3, 410.4, 410.5, 410.6, 410.7, 410.8, 410.9, 411, 411.1, 411.81, 411.89, 412, 413, 413.1, 413.9, 414
MCI	ICD-10: F067
Migraine	346, 346.1, 346.2, 346.8, 346.9
Obesity	278, 278.01
Parkinson	332, 332.1
TBI	800, 800.3, 801, 802, 802.1, 802.2, 802.3, 802.4, 802.5, 802.6, 802.7, 802.8, 802.9, 803, 803.4, 803.5, 803.9, 804, 850, 850.1, 850.2, 850.4, 850.5, 851, 851.02, 851.03, 851.04, 851.05, 851.06, 851.1, 851.12, 851.13, 851.14, 851.15, 851.8, 851.9, 852, 852.2, 852.4, 853, 854

Note. Abbreviations. AF, Atrial Fibrillation. COPD, Chronic Obstructive Pulmonary Disease. HF, Heart Failure. IHD, Ischemic Heart Disease. MCI, Mild Cognitive Impairment. TBI, Traumatic Brain Injury.

© 2023 Levine SZ et al. *JAMA Network Open.*

eTable 2. Rates per 10,000 Person-Years

Covariate	Classification	Rate (95% CI)
Sex	Female	1.49 (1.44,1.53)
Sex	Male	1.40 (1.36,1.45)
SES	High	1.60 (1.49,1.71)
SES	Low	1.31 (1.26,1.37)
SES	Medium	1.49 (1.45,1.54)
Smoker	Present	2.80 (2.64,2.96)
Smoker	Absent	1.33 (1.30,1.36)
Depression	Present	6.15 (5.71,6.62)
Depression	Absent	1.34 (1.31,1.38)
Obesity	Present	2.38 (2.28,2.49)
Obesity	Absent	1.28 (1.25,1.32)
COPD	Present	3.43 (3.23,3.64)
COPD	Absent	1.32 (1.29,1.36)
Hypertension	Present	2.55 (2.48,2.61)
Hypertension	Absent	0.56 (0.54,0.59)
AF	Present	5.16 (4.85,5.48)
AF	Absent	1.30 (1.27,1.33)
HF	Present	6.70 (6.08,7.37)
HF	Absent	1.38 (1.35,1.42)
IHD	Present	3.73 (3.57,3.89)
IHD	Absent	1.17 (1.14,1.20)
Cerebrovascular	Present	6.42 (6.16,6.68)
Cerebrovascular	Absent	1.08 (1.06,1.11)
Diabetes	Present	3.34 (3.23,3.46)
Diabetes	Absent	1.04 (1.01,1.08)
Parkinson	Present	17.21 (15.95,18.55)
Parkinson	Absent	1.33 (1.30,1.36)
TBI	Present	5.57 (4.62,6.66)
TBI	Absent	1.43 (1.40,1.46)
Migraine	Present	1.67 (1.49,1.85)
Migraine	Absent	1.44 (1.41,1.47)
MCI	Present	36.15 (32.48,40.14)
MCI	Absent	1.38 (1.35,1.42)
Psychostimulant	Present	6.58 (5.54,7.76)
Psychostimulant	Absent	1.43 (1.40,1.46)
Adult ADHD	Present	5.19 (4.20,6.34)
Adult ADHD	Absent	1.44 (1.40,1.47)

Note. Abbreviations. SES, socioeconomic status, COPD, Chronic Obstructive Pulmonary Disease. AF, Atrial Fibrillation. HF, Heart Failure. IHD, Ischemic Heart Disease. TBI, Traumatic Brain Injury MCI, Mild Cognitive Impairment., Adult ADHD, Adult Attention-Deficit/Hyperactivity Disorder 95% CI Confidence Intervals.

© 2023 Levine SZ et al. *JAMA Network Open.*

eTable 3. Cox Proportional Hazards Regression Model From the Primary Analysis With All Study Covariates

Covariate	Hazard Ratio (95% Confidence Interval)	P-value
Age 2002 (linear term)	0.85 (0.76, 0.95)	0.003
Age 2002 (quadratic term)	1.00 (1.00, 1.00)	<.001
Female Sex	1.09 (1.04, 1.15)	<.001
SES Medium	1.04 (0.99, 1.10)	0.14
SES High	1.07 (0.98, 1.16)	0.14
Smoking status	1.10 (1.02, 1.19)	0.02
Depression	2.12 (1.92, 2.35)	<.001
Obesity	1.02 (0.96, 1.09)	0.46
COPD	1.30 (1.20, 1.40)	<.001
Hypertension	1.82 (1.71, 1.95)	<.001
AF	1.29 (1.19, 1.40)	<.001
HF	1.27 (1.13, 1.43)	<.001
IHD	1.17 (1.09, 1.24)	<.001
Cerebrovascular disease	2.37 (2.23, 2.52)	<.001
Diabetes	1.58 (1.49, 1.67)	<.001
Parkinson	4.03 (3.62, 4.50)	<.001
TBI	1.74 (1.21, 2.51)	0.003
Migraine	1.04 (0.93, 1.17)	0.50
MCI	6.20 (5.18, 7.44)	<.001
ADHD medication	1.39 (1.12, 1.72)	0.003
Adult ADHD	2.77 (2.11, 3.63)	<.001

Note. Abbreviations. SES, socioeconomic status. COPD, Chronic Obstructive Pulmonary Disease. AF, Atrial Fibrillation. HF, Heart Failure. IHD, Ischemic Heart Disease. TBI, Traumatic Brain Injury. MCI, Mild Cognitive Impairment. Adult ADHD, Adult Attention-Deficit/Hyperactivity Disorder.
The reference group for sex is male sex, for SES, low SES, and from Smoking status to Adult ADHD, condition or status absent.

© 2023 Levine SZ et al. *JAMA Network Open.*

eFigure 1. Reverse Causation Illustrated

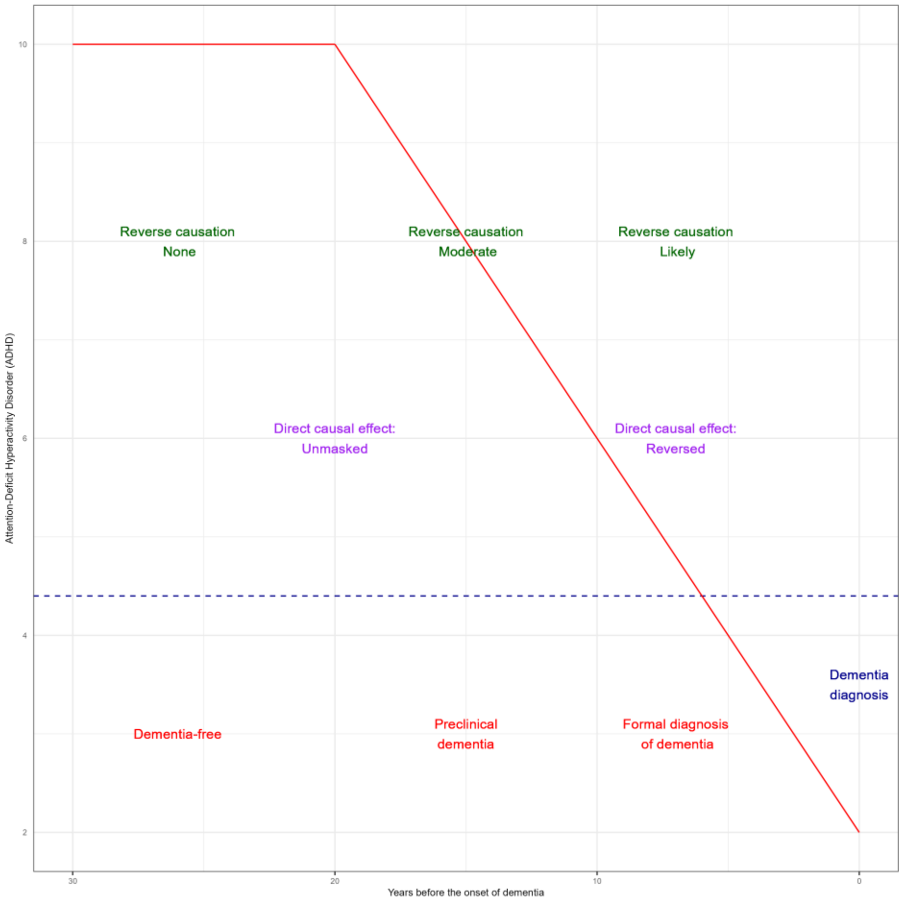

Note. eFigure 1 was adapted from Kivimäki, Luukkonen, Batty, Ferrie, Pentti, Nyberg, et al. [1].
To examine reverse causation, the analysis was stratified by sequential duration of follow-up. Based on three successive five-year intervals, the associations between ADHD and dementia risk were scrutinized. ADHD was assumed to have less effect on dementia risk in the preclinical dementia stage when the assessment was long before dementia onset and considerably affected risk when the assessment was nearer the diagnosis. Therefore, a stronger association in the first intervals of follow-up time may suggest that reverse causation occurs.

© 2023 Levine SZ et al. *JAMA Network Open.*

eFigure 2. Follow-Up Time Illustration in the Survival Analysis

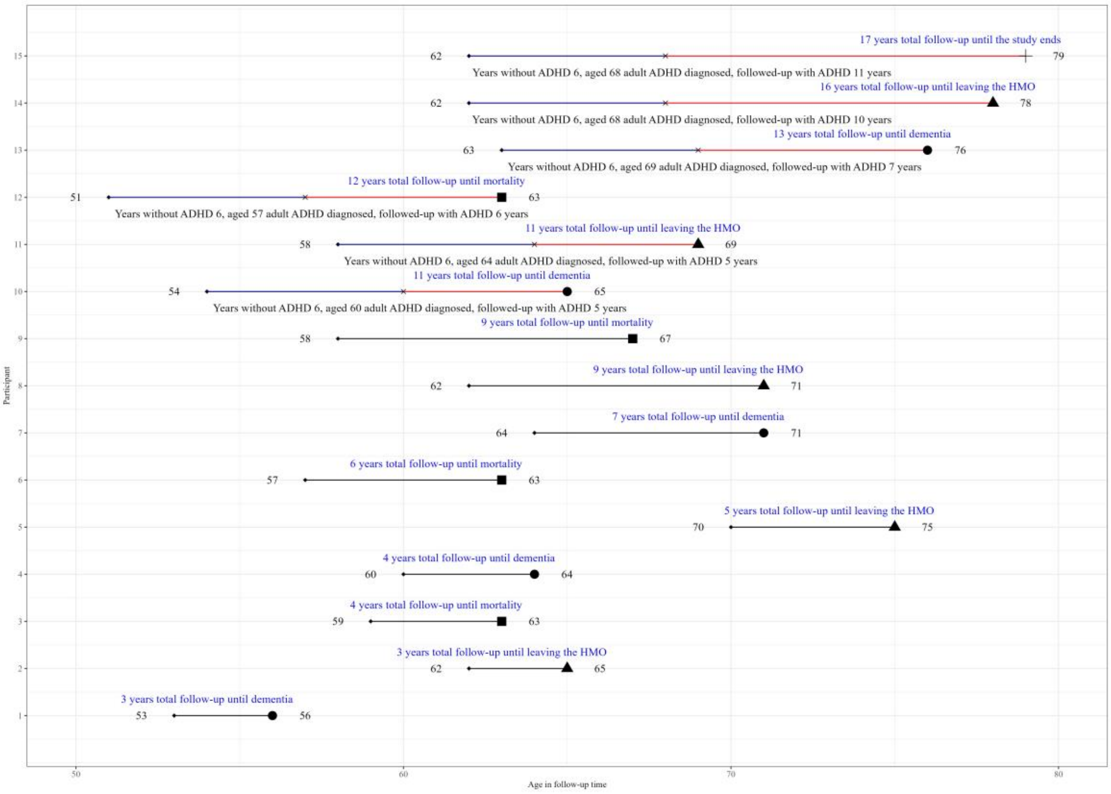

Note. eFigure 2 illustrates the cohort entry and end ages of follow-up for 15 fictitious study participants. From the left, we can observe the ages when participants entered the cohort (age and diamond) and to the far right when censored and follow-up ended. Above the line of each participant in blue is the number of years at risk with the reason follow-up ended (Triange: leaving the HMO; Square: all-cause mortality; Circle: Dementia; Cross: end of study follow-up). Participants 10 to 15 illustrate the time-varying nature of adult ADHD. For these participants, a blue colored line indicates their periods without an ADHD diagnosis, the age at adult ADHD diagnosis (indicated by a star), and a red line represents ADHD during follow-up. For instance, participant 15 began the study at age 62, was followed up for six years without adult ADHD, was diagnosed with adult ADHD at age 68, and was followed-up with adult ADHD for a further 11 years and completed the total study follow-up of 17 years at age 79 without dementia or mortality. The remaining participants in eFigure 2 were followed-up to dementia, death, or leaving the HMO during follow-up, and so were followed up for less time.

© 2023 Levine SZ et al. *JAMA Network Open.*

eFigure 3. Cumulative Incidence Plot

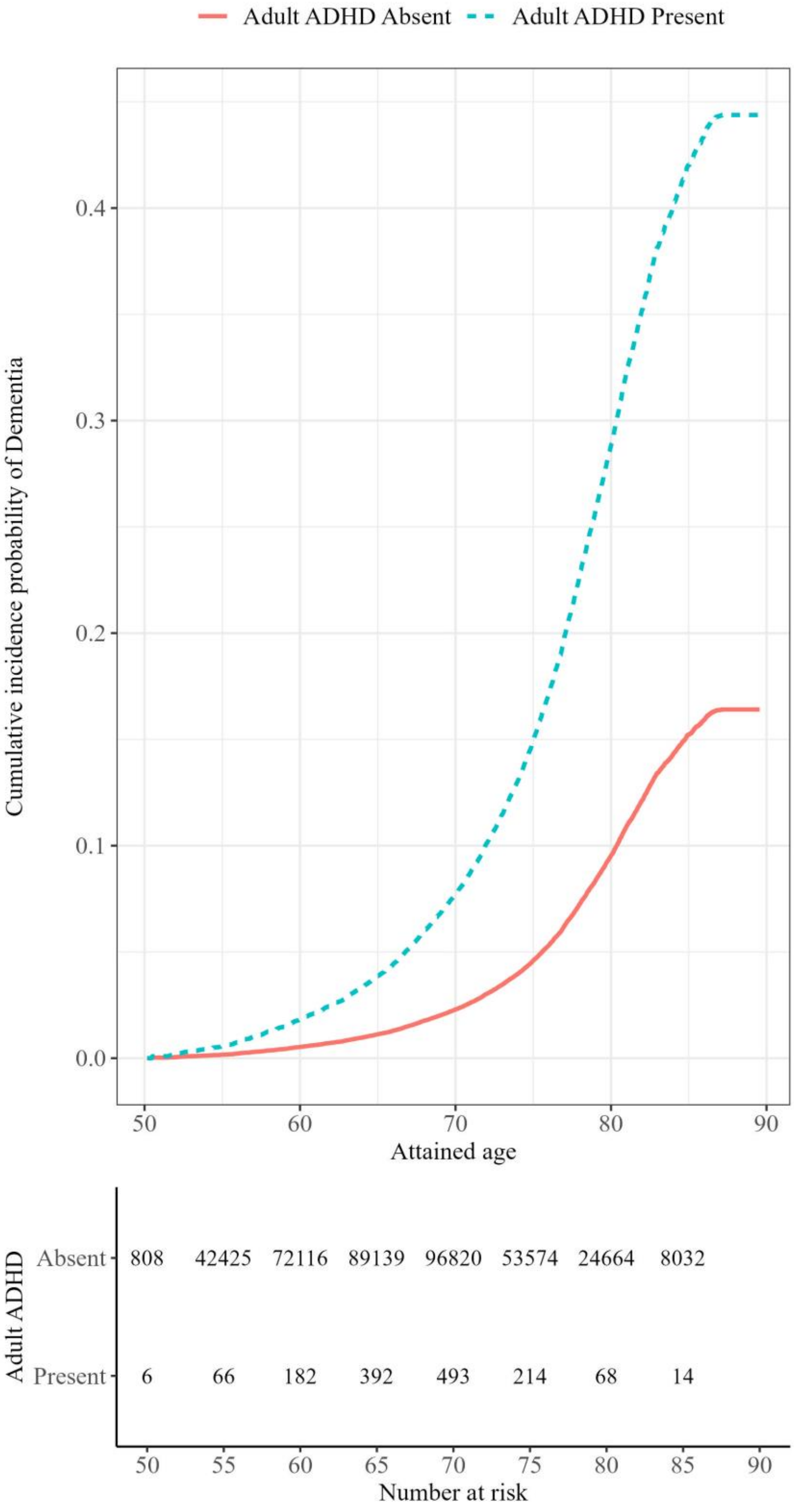

Note. The cumulative incidence probability was estimated using the inverse survival plot function from the unadjusted Cox regression model.[2] Under the curves are the number at-risk by adult ADHD status. As shown in eFigure 3, individuals enter the ADHD cohort at different ages (lowest section). So, for example, an individual entering the study cohort ADHD-free at age 55, diagnosed with adult ADHD at age 65, and censored (for dementia, death, or leaving the HMO) at age 70 contributes to ten years of risk time to the adult ADHD-absent group and five years of risk time to the adult ADHD present group.

© 2023 Levine SZ et al. *JAMA Network Open.*

eReferences.

1. Kivimäki M, Luukkonen R, Batty GD, et al. Body mass index and risk of dementia: Analysis of individual-level data from 1.3 million individuals. *Alzheimers Dement.* 2018;14(5):601-609.
2. Therneau TM, Grambsch PM. *Modeling Survival Data: Extending the Cox Model.* New York, NY: Springer; 2000.

© 2023 Levine SZ et al. *JAMA Network Open.*
