# Supplementary material for: Adult Attention-Deficit/Hyperactivity Disorder and the Risk of Dementia
Source: JAMA Netw Open. 2023 Oct 17;6(10):e2338088. doi: 10.1001/jamanetworkopen.2023.38088 (PMC10582792; doi:10.1001/jamanetworkopen.2023.38088)
Supplement: Supplement 2. — Data Sharing Statement [file jamanetwopen-e2338088-s002.pdf]

Data Sharing Statement

Levine. Adult Attention-Deficit/Hyperactivity Disorder and the Risk of Dementia. *JAMA Netw Open*. Published October 17, 2023. doi:10.1001/jamanetworkopen.2023.38088

Data
Data available: No

Additional Information
Explanation for why data not available: Due to data security and privacy regrettably we are unable to make these data that are from electronic healthcare records available.
